# Masticación de la hoja de coca como factor de riesgo para la salud bucal

**DOI:** 10.15446/rsap.V25n5.109421

**Published:** 2023-09-01

**Authors:** Linda P. Lucas-Espeza, Ronald de la Cruz-Rodríguez, Elias E. Aguirre-Siancas

**Affiliations:** 1 LE: Cir. Dent. M. Sc. Docencia e Investigación en Salud, Universidad Nacional Mayor de San Marcos. Lima, Perú. linda.lucas@upch.pe Universidad Nacional Mayor de San Marcos Investigación en Salud Universidad Nacional Mayor de San Marcos Lima Peru linda.lucas@upch.pe; 2 RC: OD. Universidad Nacional Mayor de San Marcos. Lima, Perú. ronaldalexisdelacruzrodriguez@gmail.com Universidad Nacional Mayor de San Marcos Universidad Nacional Mayor de San Marcos Lima Peru; 3 EA: Cir. Dent. Ph. D. Neurociencias, Universidad Nacional Mayor de San Marcos. Lima, Perú. eaguirres@unmsm.edu.pe Universidad Nacional Mayor de San Marcos Universidad Nacional Mayor de San Marcos Lima Peru eaguirres@unmsm.edu.pe

**Keywords:** Coca, salud bucal, gingivitis, periodontitis, articulación temporomandibular, músculos faciales *(fuente: DeCS, BIREME)*, Coca, oral health, gingivitis, periodontitis, temporomandibular joint, facial muscles *(source: MeSH, NLM)*

## Abstract

**Objetivo:**

Determinar si la masticación de la hoja de coca es un factor de riesgo para la salud bucal.

**Método:**

Estudio de casos y controles desarrollado en el centro de salud Paucará, Huancavelica (Perú), en el año 2019. La muestra estuvo conformada por 200 participantes, divididos en dos grupos: 100 consumidores de hoja de coca y 100 no consumidores. Para evaluar la salud bucal, se empleó un instrumento validado por cinco expertos, el cual tuvo en cuenta tres puntos: articulación temporomandibular, cavidad oral y características de la masticación de la hoja de coca.

**Resultados:**

En la población estudiada se identificó que el género femenino es el mayor consumidor de la hoja de coca. Además, se observó que dicho consumo no produce desgaste, fractura o movilidad dentaria; sin embargo, genera alteración de la encía (OR:42,67). Según la evaluación del dolor muscular, se pudo observar que la masticación de la hoja de coca afectó significativamente al músculo masetero, inserción derecha, en comparación con aquellos individuos que no practican esta actividad (OR:17,47). Por otra parte, se encontró que los movimientos mandibulares alterados y los ruidos articulares en ambos grupos no se asocian con alteración de la articulación temporomandibular.

**Conclusión:**

La masticación de la hoja de coca afecta significativamente la encía y el músculo masetero inserción derecha, en comparación con no masticarla.

## Abstract (en)

**Objective:**

To determine whether coca leaf chewing is a risk factor for oral health.

**Methods:**

Case-control study carried out in the Paucará health centre, Huancavelica 2019. The sample consisted of 200 participants, divided into two groups: 100 coca leaf users and 100 non-coca leaf users. To assess oral health, an instrument validated by five experts was used, where three items were evaluated: Temporomandibular joint, oral cavity and coca leaf chewing characteristics.

**Results:**

In the population studied, it was identified that the female gender is the big-gest consumer of coca leaf. In addition, it was observed that coca leaf consumption does not produce dental wear, fracture or mobility; however, its consumption gene-rates alteration of the gum (OR:42.67). According to the evaluation of muscle pain, it was observed that coca leaf chewing significantly affected the right insertion masseter muscle, compared to those who did not consume it (OR:17.47). Moreover, it was found that altered jaw movements and joint noises in both groups were not associated with temporomandibular joint disturbance.

**Conclusion:**

Coca leaf chewing significantly affects the gum and masseter muscle right Insertion compared to non-coca leaf chewers.

La hoja de coca (HC), científicamente denominado *Erythroxylum coca,* es una planta utilizada por los pobladores de los Andes, que se cultiva en zonas cálidas de la selva amazónica. Su nombre proviene del término aimara *Kkoka,* que significa "planta divina". Una de las razones del consumo es aumentar la energía física y mental, ya que se considera un estimulante natural 1.

En el Perú, alrededor tres millones de individuos consumen HC cada año, lo que equivale a un consumo total de aproximadamente 7 500 toneladas. Del total de consumidores, la mayoría reside en la región de la sierra del Perú, y se consideran masticadores habituales a aquellos que mastican la coca entre una y siete veces a la semana 2.

El consumo frecuente de HC puede perjudicar la salud bucal, al causar alteraciones en la articulación temporomandibular (ATM), desgaste patológico en la estructura de las piezas dentarias y alteración de las encías. Muchos consumidores añaden cal (sustancia alcalina) al masticar las hojas, lo cual varia el pH salival y se relacionaría con un mayor daño en la salud bucal 3.

En un estudio realizado por Valeriano 4, se determinó que los consumidores de HC presentan leucoplasia, inflamación moderada de la encía, cálculo supra y subgingival, aftas bucales y mayor pigmentación melánica Por su parte, Ayon y Chu 5 encontraron que los masticadores de HC presentaban alteraciones de las características histológicas de la mucosa gingival, significativas a nivel del tejido epitelial, con mayor presencia de acantosis e hiperqueratosis, en comparación con los no masticadores. En contrapartida, algunos estudios, como el de Ordinola 6, al evaluar la asociación de la enfermedad periodontal con la masticación de HC, encontraron que no había asociación estadísticamente significativa entre ambas variables.

A pesar de que la relación de las variables propuestas constituye un problema de salud pública, sobre todo en regiones rurales, está relación ha sido poco estudiada y como consecuencia escasamente documentada en artículos científicos. El objetivo de este estudio fue determinar si la masticación de la HC es un factor de riesgo para la salud bucal, con alteraciones clínicas de la cavidad oral y de la articulación temporomandibular en personas de 20 a 49 años.

## MATERIALES Y MÉTODOS

### Diseño y población

Se hizo un estudio observacional, analítico, de tipo de casos y controles. Los casos fueron los pacientes masticadores de hoja de coca y los controles los pacientes que no tenían dicho hábito. La población estuvo conformada por personas de 20 a 49 años que acudieron al servicio de odontología del centro de salud Paucará en el distrito Paucará, provincia de Acobamba, departamento de Huancavelica en la sierra del Perú, entre los meses de septiembre del 2018 y febrero del 2019. Se analizaron las características del consumo de HC, la cavidad bucal y la ATM de cada paciente, con base en el instrumento validado por Lucas 7.

### Muestra

El tamaño de la muestra se determinó mediante la fórmula para un estudio de casos y controles, teniendo en cuenta los siguientes parámetros: *Z*_*1*_ *-x/2:* nivel de confianza del 95 %, con valor de 1,96; *Z*_*1*_ *-b:* nivel de poder estadístico del 80 %, con valor de 0,84; *P*_*1*_ *:* frecuencia de exposición protrusión máxima entre los casos, según piloto, con valor de 0,3. *P*_*2*_ *:* frecuencia de exposición protrusión máxima entre los controles, según piloto, con valor de 0,1; P: prevalencia de estudio, con valor de 0,2 (7). Luego de aplicar la fórmula se obtuvo que se necesita a 84 personas con masticación de HC y a 84 sin tal masticación; sin embargo, la presente investigación se realizó con 100 personas por cada grupo, reclutadas en los meses indicados en el párrafo previo.

### Criterios de selección de la muestra

Tanto los casos como los controles firmaron un consentimiento informado, además, todos fueron pacientes del centro de salud de Paucará. Dentro de los masticadores de HC se incluyó a aquellos que indicaban ser mastica-dores habituales, con un tiempo mínimo de seis años de hábito. Y entre los no masticadores se incluyó a aquellos que en la entrevista previa señalaban que nunca habían masticado HC. De ambos grupos de la muestra se excluyó a las personas con traumatismos a nivel de los músculos masticatorios y que padecieran artritis reumatoide, fibromialgia o traumatismo mandibular, o que hubiesen estado o estuvieran bajo tratamiento ortodóntico. Tanto para los casos como para los controles se mantuvo una proporción similar de pacientes masculinos y femeninos.

### Técnica de recolección de datos

La entrevista y el examen clínico se llevaron a cabo en el servicio de odontología del centro de salud Paucará. La evaluación de la cavidad bucal, en la que se incluyeron los dientes y la mucosa oral, se realizó empezando por la parte posterior derecha del maxilar (cuadrante 1) hasta llegar a la mucosa y a los dientes finales de la parte izquierda maxilar (cuadrante 2); luego se continuó con la evaluación de la mandíbula (cuadrante 3 y 4). En el análisis del ATM se evaluaron los movimientos mandibulares, el desplazamiento del cóndilo y los músculos faciales, para lo cual se empleó la técnica descrita por Lucas 7.

En la evaluación de la masticación de la HC se preguntó sobre el uso de cal, la frecuencia y otras características de consumo con en base el instrumento de Lucas 7.

### Validación del instrumento empleado

El instrumento, validado por cinco expertos en cirugía maxilofacial, consta de tres puntos: articulación temporomandibular, cavidad oral y masticación de la HC. Los cuestionarios de la articulación temporomandibular se subdividen en escala del movimiento mandibular basado en el índice de Helkimo 8 y desplazamiento del cóndilo que pertenece al criterio diagnóstico (CDI/TTM eje I), descritos por Dworkin y LeResche 9. Los ítems de cavidad oral y masticación de la *HC* fueron añadidos por criterios propios, con base en una extensa revisión de la literatura. Se validó un total de 38 ítems del instrumento mediante el empleo de la V de Aiken, y se obtuvo una validez fuerte de 0,93. La confiablidad se determinó con el coeficiente de Alfa de Cronbach, siendo buena con un valor de 0,830 7.

Como variables independientes se tuvieron *masticación de la hoja de coca* y *no masticación de la hoja de coca.* La variable dependiente fue la salud bucal, cuyas dimensiones fueron la articulación temporomandibular y la cavidad oral.

### Análisis estadístico

Para el análisis y la interpretación de los datos se empleó el programa SPSS 21 y el programa Excel 2016. Los datos se presentan en tablas de frecuencia, organizados en sus valores absolutos y en valores porcentuales. Para evaluar la asociación entre las variables propuestas se empleó Odds Ratio (OR) y se indicaron sus respectivos intervalos de confianza al 95%.

### Aspectos éticos

El protocolo del estudio contó con la aprobación del Comité de Ética de la Facultad de Medicina de la Universidad Nacional Mayor de San Marcos, con acta de aprobación N.° 0448.

## RESULTADOS

En el estudio participaron en total 168 mujeres (84%) y 32 varones (16%). Se encontró que 12 (37,5%) varones y 88 (52,4%) mujeres mastican HC. A su vez, 60 participantes utilizan la cal como un aditivo en el consumo de HC, en tanto que 40 no la utilizan. Asimismo, 45 personas consumieron HC más cal una a dos veces por semana, 12 consumieron HC más cal de tres a seis veces por semana, y tres personas consumieron HC más cal de siete a más veces por semana.

### Resultados descriptivos

Se evaluaron varias estructuras de la cavidad oral, como la encía, la fractura limitada al esmalte y dentina sin exposición pulpar, movilidad dentaria y superficie desgastada, como se describe en la Tabla 1.

**Tabla 1:** Distribución de las personas con y sin masticación de coca de acuerdo con hallazgos encontrados en la cavidad oral

Cavidad oral		Masticación de hoja de coca		Total	
Estructura evaluada	Indicador	Si	No	N	%
Encía	Sana	36	96	132	66,0
	Con gingivitis	50	4	54	27,0
	Con periodontitis	14	0	14	7,0
Fractura limitada al esmalte y dentina sin exposición pulpar	Presente	3	0	3	1,5
	Ausente	97	100	197	98,5
Movilidad dentaria	Ninguno	88	100	188	94,0
	Menos de 1mm de movimiento horizontal	12	0	12	6,0
Superficie desgastada	Sin desgaste	22	100	122	61,0
	Pérdida de esmalte	64	0	64	32,0
	Pérdida de esmalte*	14	0	14	7,0

* Con exposición de menos de un tercio de la dentina.

**Figura 1:**
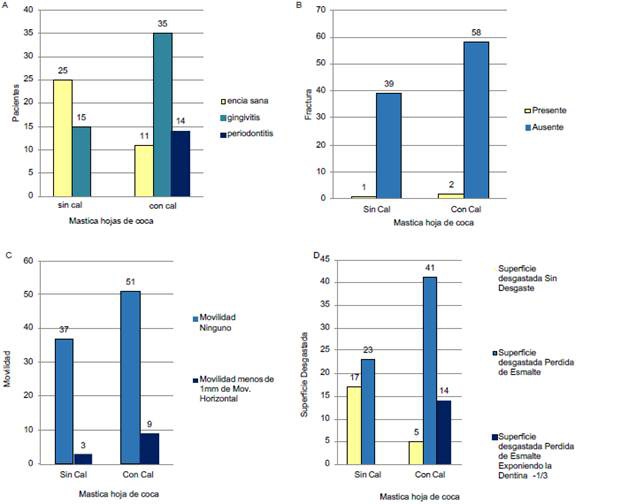
Collage de las cuatro imágenes: Evaluación de la masticación de uso de coca con uso y sin uso de cal sobre la cavidad oral. El collage debería queda así:

En la Figura 1 se presentan los resultados de la evaluación de la encía, la fractura dentaria, la movilidad dentaria y la superficie desgastada en las personas que consumieron específicamente HC con uso y sin uso de cal.

En el trabajo se analizaron varias funciones musculares, como se presenta en la Tabla 2. Así, se evaluaron los principales movimientos mandibulares dependientes de la articulación temporomandibular, tales como la apertura máxima, la lateralidad derecha máxima, la lateralidad izquierda máxima y la protrusión máxima.

**Tabla 2:** Evaluación de los movimientos mandibulares dependientes de la atm en las personas con y sin masticación de la hoja de coca

Movimiento evaluado	Centímetros	Masticación de hoja de coca		Total	
		Si	No	N	%
Apertura máxima	≥ 40	0	99	99	49,5
	30 - 39	89	1	90	45,0
	< 30	11	0	11	5,5
Lateralidad derecha máxima	≥ 7	0	100	100	50,0
	4- 6	95	0	95	47,5
	< 4	5	0	5	2,5
Lateralidad izquierda máxima	≥ 7	0	100	100	50,0
	4- 6	100	0	100	50,0
Protrusión máxima	≥ 7	0	100	100	50,0
	4- 6	93	0	93	46,5
	< 4	7	0	7	3,5

Los ruidos articulares, como el chasquido o la crepitación, fueron evaluados por los desplazamientos del cóndilo de la articulación temporomandibular en los consumidores y en los no consumidores de HC. Dicha evaluación se realizó con base en los movimientos del ATM en: apertura bucal derecha, apertura bucal izquierda, cierre derecho e izquierdo y excursión derecha e izquierda, como se muestra en la Tabla 3. Se observa que no se encontraron ruidos articulares en los no consumidores de HC; sin embargo, si se hallaron dichos ruidos en los masticadores de HC.

**Tabla 3:** Evaluación de los desplazamientos del cóndilo de la atm y el ruido articular provocado en personas con y sin masticación de la hc

Movimiento evaluado	Ruido encontrado	Masticación de hoja de coca		Total	
		Si	No	N	%
Apertura derecha	Ninguno	89	100	189	94,5
	Chasquido	11	0	11	5,5
	Bastante crepitación	1	0	1	0,5
Apertura izquierda	Ninguno	96	100	196	98,0
	Chasquido	4	0	4	2,0
Cierre derecho	Ninguno	93	100	193	96,5
	Chasquido	7	0	7	3,5
Cierre izquierdo	Ninguno	91	100	191	95,5
	Chasquido	9	0	9	4,5
Excursión derecha	Ninguno	93	100	193	96,5
	Chasquido	7	0	7	3,5
Excursión izquierda	Ninguno	95	100	195	97,5
	Chasquido	5	0	5	2,5

De acuerdo con los resultados de la evaluación realizada para cada músculo facial en las personas masticadoras de HC *vs.* las no consumidoras, se encontró que los masti cadores presentan diferentes frecuencias de dolor en todos los casos. En contraste, solo se encontró dolor en el músculo masetero inserción derecha en las personas no masticadoras de HC. Los detalles de las evaluaciones se muestran en la Tabla 4.

**Tabla 4:** Evaluación del dolor muscular relacionado con la función de la articulación temporomandibular

Porción muscular evaluada	Dolor	Masticación de hoja de coca		Total	
		Si	No	N	%
	Ninguno	93	100	193	96,5
Músculo temporal post derecho	Leve	6	0	6	3,0
	Moderado	1	0	1	0,5
Músculo temporal post izquierdo	Ninguno	96	100	196	98,0
	Leve	4	0	4	2,0
Musculo temporal medio derecho	Ninguno	94	100	194	97,0
	Leve	6	0	6	3,0
Músculo temporal medio izquierdo	Ninguno	96	100	196	98,0
	Leve	4	0	4	2,0
Músculo temporal anterior derecho	Ninguno	96	100	196	98,0
	Leve	4	0	4	2,0
Músculo temporal anterior izquierdo	Ninguno	95	100	95	97,5
	Leve	5	0	5	2,5
Músculo masetero cuerpo derecho	Ninguno	97	100	197	98,5
	Leve	3	0	3	1,5
Músculo masetero cuerpo izquierdo	Ninguno	98	100	198	99,0
	Leve	2	0	2	1,0
Músculo masetero inserción derecha	Ninguno	85	99	184	92,0
	Leve	15	1	16	8,0
Músculo masetero inserción izquierdo	Ninguno	98	100	198	99,0
	Leve	2	0	2	1,0
Músculo masetero origen derecho	Ninguno	97	100	197	98,5
	Leve	3	0	3	1,5
Músculo masetero origen izquierdo	Ninguno	97	100	197	98,5
	Leve	3	0	3	1,5
Región del esternocleidomastoideo derecho	Ninguno	96	100	196	98,0
	Leve	4	0	4	2,0
Región del esternocleidomastoideo izquierdo	Ninguno	98	100	198	99,0
	Leve	2	0	2	1,0
Región anterior del digástrico derecho	Ninguno	97	100	197	98,5
	Leve	3	0	3	1,5
Región anterior del digástrico izquierdo	Ninguno	92	100	192	96,0
	Leve	8	0	8	4,0
Músculo pterigoideo lateral derecho	Ninguno	96	100	196	98,0
	Leve	4	0	4	2,0
Músculo pterigoideo lateral izquierdo	Ninguno	97	100	197	98,5
	Leve	3	0	3	1,5
Tendón del temporal derecho	Ninguno	97	100	197	98,5

### Resultados inferenciales

Para evaluar la asociación entre la masticación o no de HC y todos los indicadores presentados, tanto de la cavidad bucal como del ATM, se empleó la prueba de Odds Ratio. De acuerdo con los resultados de dicha prueba, se determinó que una persona que mastica HC tiene 42,67 veces más riesgo de presentar algún tipo de alteración en la encía, en comparación con las personas que no la consumen (011=42,67, IC 95% 14,49-125,68). Asimismo, se encontró que las personas que mastican HC tienen 17,47 veces más riesgo de presentar dolor del músculo masetero inserción derecha, comparado con las personas que no la mastican (0R = 17,47, IC 95% 2,26-135,02).

En el resto de las evaluaciones no se encontró asociación con base en la prueba de OR, debido a que los pacientes no masticadores de HC no presentaron alteración.

## DISCUSIÓN

Los resultados del presente estudio muestran que la mayoría de los consumidores de HC son del sexo femenino (52,4%); además, la mayor frecuencia de consumo es de una a dos veces por semana, acompañado del uso de cal. Por otro lado, las personas que mascan HC presentan más riesgo de alteración en las encías y dolor del músculo masetero inserción derecha.

Ordinola *et al.*^6^ estudiaron la asociación entre la gingivitis y la periodontitis con el consumo de HC más cal en 80 trabajadores masculinos de 19 a 70 años. Dicho estudio no encontró una asociación significativa entre las variables evaluadas. Nuestros resultados difieren de dicha investigación, ya que hemos encontrado que un masticador de HC tiene 42,67 veces más riesgo de padecer alteración de la encía. Esta diferencia podría deberse a que la muestra en el estudio citado solo estuvo conformada por varones, a diferencia del nuestro donde 88 % de los masticadores eran mujeres. Es conocido que los cambios hormonales cíclicos femeninos, unidos a la masticación de HC y una pobre higiene oral, predisponen a las mujeres a padecer diversos grados de gingivitis 10, a diferencia de los varones, que no tienen dicha fisiología hormonal. Además, Valeriano 4 investigó la salud oral en 65 personas mayores de 18 años y masticadoras de HC, y encontró inflamación moderada de la encía en un 87,7%, inflamación severa de la encía en 6,2%, presencia de cálculo supra y subgingival en 49,2%, aftas bucales en 24,6%, leucoplasia en 3,1% y sangrado después del sondeo en 38,5%. Dicho estudio coincide con la presente investigación en los niveles de inflamación de las encías, aunque el trabajo citado evaluó muchos más indicadores que los que nuestro estudio realizó.

Chura 11 estudió la relación entre trastornos temporomandibulares y masticación de HC en 158 personas, de las cuales 110 fueron mujeres y el resto varones, y encontró una correlación directa entre ambas variables, de acuerdo con el índice de Helkimo (p<0,001), con una prevalencia de 75,3 % y siendo más frecuente en el sexo femenino. Un trabajo similar, llevado a cabo por Angulo 12, estudió la frecuencia de los trastornos temporomandibulares en masticadores de HC en 95 personas, de los cuales 81 fueron varones y 14 mujeres, y encontró que la prevalencia de trastornos temporomandibulares fue de 89,5% (varones 93,8 % y mujeres 64,3 %). En nuestro trabajo, no encontramos asociación entre alteraciones en la ATM y masticación de HC; solo se determinó puntualmente que los masticadores de HC tienen 17,47 veces más riesgo de presentar dolor del músculo masetero inserción derecha que aquellos que no mastican.

Por otra parte, al evaluar las estructuras dentarias, se encontró que una minoría presentó fractura y movilidad dentaria, con una alta prevalencia (78 %) al evaluar las superficies dentales desgastadas de esmalte y de esmalte-dentina en los masticadores de HC. Sin embargo, al hacer las inferencias estadísticas, dicha prevalencia no se asoció con la masticación de HC. También es oportuno resultar que no se han encontrado antecedentes en la literatura científica que hallan evaluado la relación entre la masticación de HC y, puntualmente, las alteraciones dentarias.

En conclusión, se observó que el sexo femenino tiene una mayor tendencia a consumir HC, en tanto que la mayor frecuencia de consumo es de una a dos veces por semana. El hábito de masticar HC con o sin cal, no origina alteraciones de desgaste dentario, fractura o movilidad dentaria, al no encontrarse relación significativa. Sin embargo, las personas consumidoras de HC tienen mayor riesgo de presentar alteración de la encía y dolor del músculo masetero inserción derecha. Por otro lado, no se ha encontrado una relación significativa entre los movimientos mandibulares alterados y los ruidos articulares con el consumo de HC.

Entre las limitaciones del trabajo se puede mencionar que algunos pacientes solo entendían quechua, para ellos se contó con apoyo de personal del centro de salud quechua hablante como traductores; no obstante, algunos pacientes quechua hablantes no aceptaron participar del trabajo por dicha limitación idiomática. Por otra parte, hubiese sido adecuado tomar radiografías u otras pruebas imagenológicas para evaluar la ATM y las estructuras óseas, pero el centro no contaba con tales equipos. Se recomienda seguir investigando la asociación entre las variables propuestas en distintas regiones del Perú y países latinoamericanos donde esté arraigado el hábito, de manera que se fomenten programas de atención y sensibilización sobre la masticación de la hoja de coca y sus perjudiciales efectos sobre la salud bucal. Además, en futuros estudios se recomienda emplear exámenes imagenológicos ♣
